# Blue honeysuckle seeds and seed oil: Composition, physicochemical properties, fatty acid profile, volatile components, and antioxidant capacity

**DOI:** 10.1016/j.fochx.2024.101176

**Published:** 2024-02-07

**Authors:** Juan Sun, Dalong Li, Wenjing Huyan, Xiaoqi Hong, Shuman He, Junwei Huo, Lianzhou Jiang, Yan Zhang

**Affiliations:** a Heilongjiang Green Food Science Research Institute, Northeast Agricultural University, Harbin 150030, China; b Key Laboratory of Biology and Genetic Improvement of Horticultural Crops (Northeast Region), Ministry of Agriculture and Rural Affairs, Northeast Agricultural University, Harbin 150030, China; c National-Local Joint Engineering Research Center for Development and Utilization of Small Fruits in Cold Regions, Northeast Agricultural University, Harbin 150030, China; d College of Horticulture and Landscape Architecture, Northeast Agricultural University, Harbin 150030, China; e College of Food Science, Northeast Agricultural University, Harbin 150030, China

**Keywords:** Haskap seeds, Seed oil, Volatile compounds, Fatty acids, GC–MS

## Abstract

Blue honeysuckle seeds are often overlooked by the processing industry, but they are a good source of healthy oil. The composition, volatiles, and antioxidant capacity of blue honeysuckle seeds and seed oil were investigated for the first time. The fatty acid profile of the seed oil was analysed using GC–MS. The seed oil was particularly rich in polyunsaturated fatty acid, especially linoleic acid (71.24 ± 1.64 %). HS-SPME-GC–MS analysis temporarily detected 34 and 37 volatiles in the seeds and seed oil, respectively. Notably, aldehydes were identified as the major contributors to the aroma. The phytosterols, tocopherols, and triglycerides were identified in the seed oil. Interestingly, the total phenolic content and antioxidant capacity of the seeds were found to be much higher than the seed oil. This study evaluates the nutritional profile and value of blue honeysuckle seed oil, and suggests that it can be used as new functional oil.

## Highlights

• This study marks the first investigation into the seeds and seed oil of blue honeysuckle.
• The seed oil contained 95.94% unsaturated fatty acids out of the total fatty acids.
• Polyunsaturated fatty acid (specifically, linoleic acid) made up 71.24% of the seed oil.
• The seeds and seed oil were found to contain 34 and 37 volatile components, respectively.
• The seeds of blue honeysuckle exhibited a higher antioxidant capacity than the seed oil.

## Graphical abstract

**Figure:**
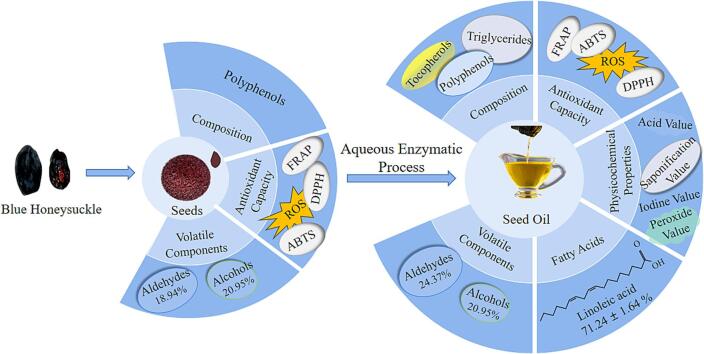

## Introduction

The blue honeysuckle (*Lonicera caerulea* L.) is a hardy, long-lived shrub in the Lonicera family, which naturally grows in severely cold regions of Russia, China, and Japan. As of 2023, the cultivation area of blue honeysuckle plants in China is about 5500 ha, and the annual fruit yield production of trees over 5 years old can reach about 40,000 tons during the high-yielding years. Heilongjiang Province is the largest blue honeysuckle plant production area in China, with the largest blue honeysuckle germplasm resources. Its dark blue fruits contain tiny seeds and are rich in anthocyanins, polysaccharides, and vitamins, with recent studies showing a link between the consumption of blue honeysuckle and a decreased risk of diseases such as cardiovascular and inflammatory disorders, diabetes, and arteriosclerosis (Cheng et al., 2022). Due to its “super fruit” status, blue honeysuckle is popularly used for fresh food, as well as for making wine, sauces, juice, and cans. Neglected fruit seeds have been found to be rich in carotenoids, polyphenols, phytosterols, and tocopherols, providing opportunities to create new value from them from an economic and nutritional perspective.

In recent years, the healthful effects of berry seed oil have garnered increasing interest, with previous research showing that berry seed oil is an excellent source of edible oil, with an oil production of about 7.6–23 % (Gustinelli, Eliasson, Svelander, Alminger, & Ahrné, 2018). Berry seed oil contains bioactive components, making it an extraordinary edible oil. Grape seeds and seed oil possess a great number of polyphenols, vitamin E, and phytosterols, and berry seed oil has a high content of unsaturated fatty acids (UFA), particularly polyunsaturated fatty acids (PUFA) (Göktürk Baydar, Özkan, & Yaşar, 2007). The grape seed oil contains a high percentage of UFA, with up to 90 % consisting mainly of linoleic acid and oleic acid (Bail, Stuebiger, Krist, Unterweger, & Buchbauer, 2008). Previous studies have also demonstrated that blackberry, black raspberry, and blueberry seed oils are rich in linoleic acid (Li, Wang, & Shahidi, 2016). Although higher PUFA content can cause oil oxidation, polyphenols, and tocopherol act as natural antioxidants, protecting the PUFA from oxidation (Van Hoed et al., 2009). However, there is limited information on the nutritional value and biological activity of blue honeysuckle seeds and seed oil.

Enzyme-assisted aqueous extraction process (EAEP) and traditional hexane extraction method are two common methods for extracting oil from seeds. The EAEP has the advantage of being a green and sustainable process, as it does not use organic solvents, which can be harmful to both human health and the environment. Additionally, EAEP can produce high-quality and healthy oil with a better nutritional profile due to the mild extraction conditions, which preserve the natural flavor and bioactive compounds of the oil. On the other hand, the traditional hexane extraction method is faster and more efficient, as it can extract a higher yield of oil compared to EAEP. However, traditional hexane extraction method has several drawbacks, such as the use of hazardous solvents, which requires special safety precautions, and the potential loss of heat-sensitive bioactive compounds due to the high temperature used during extraction (Li, Zhang, Wang, Jiang, & Sui, 2013). Therefore, the choice of extraction method should consider both the desired quality and quantity of the oil and the potential health and environmental impacts. We adopted the more environmentally friendly and healthy extraction method in order to better evaluate the phenolic contents, antioxidant capacity, volatile components, and physicochemical properties of blue honeysuckle seed oil.

While taste and quantity are the primary objectives of berries and processed products, the aroma is also crucial for success in the marketplace. Many berries with unique and complex aroma characteristics are used in processing industries such as seed oil, wine, and cosmetics production. The main aroma of blueberry fruits and seeds is associated with “green” and “grassy” aldehydes and alcohols (Bederska-Łojewska et al., 2021). Grape seed oil has a large number of alcohols, esters, ketones, and aldehydes, with nonanal as the key aroma component, providing a unique vinous and fruity aroma (Sevindik et al., 2021). However, the aroma characteristics of blue honeysuckle seeds and seed oil have not been studied. Therefore, HS-SPME-GC–MS was employed to tentatively analyse various volatile components in blue honeysuckle seeds and seed oil, so as to appreciate their distinctive characteristics. This study aims to (1) determine the contents of phenolic compound of blue honeysuckle seeds and their antioxidant capacity; (2) describe the physicochemical properties, antioxidant capacity, and bioactives such as phenolic compounds, tocopherols, triglycerides, and phytosterols in blue honeysuckle seed oil; (3) quantify and characterize the fatty acids in blue honeysuckle seed oil; and (4) systematically investigate the variations in volatile components between blue honeysuckle seeds and seed oil.

## Materials and methods

### Materials

The ripe fruit of blue honeysuckle ‘Berel’ were sampled from Northeast Agricultural University (located at 45°44′25.18″N, 126°43′22.50″E, 127.95 m above the sea level) in Harbin, Heilongjiang province, China on June 15, 2020. To ensure the reproducibility and verifiability of the research, the blue honeysuckle ‘Berel’ collected were labelled and preserved with a voucher specimen number (NEAU-Berel2011037-20200615-HY). The number contains information on the collection location, variety and Heilongjiang Province Crop Variety Registration Number of blue honeysuckle, collection date, and collector. The soil is mostly nutrient-rich black soil. The annual average temperature is 5.6 °C, with the highest monthly average temperature of 23.6 °C and the lowest monthly average temperature of −15.8 °C, and the annual average precipitation is 423 mm, mainly concentrated from June to September (Source of Data: Harbin Municipal People's Government). Blue honeysuckle ‘Berel’ is a hybrid resulting from a cross between the ‘12-19′ line (*Lonicera caerulea* subsp. *altaica*) and mixed pollen from ‘Blue spindle’, ‘Blue bird’, and ‘Azure’ varieties (Fig. S1).

Cyanidin-3-O-glucoside (C3G, purity >99 %) was purchased from Anpel Co. Ltd. (Shanghai, China). Catechin (≥98 %) was purchased from Solarbio Co. Ltd. (Beijing, China). Alcalase 2.4 L, DPPH, ABTS, TPTZ, Trolox, potassium persulfate, potassium dihydrogen phosphate, dipotassium hydrogen phosphate, gallic acid, Folin-Ciocalteu’s reagent, and α-, β-, γ- and δ-tocopherol standards were purchased from Sigma-Aldrich (St. Louis, MO, USA). Hydrochloric acid (37 %), formic acid, ethyl acetate, and HPLC grade solvents were purchased from Kermel Co. Ltd. (Tianjin, China).

### Extraction of blue honeysuckle seeds and seed oil

The seed recovery rate of the blue honeysuckle ‘Berel’ is about 2 %. The seed oil from blue honeysuckle seeds was extracted using the method of EAEP as reported in our previous study with slight modifications (Li, Zhang, Wang, Jiang, & Sui, 2013). The seeds were ground into fine powder and mixed with distilled water in a solid: water ratio of 1:4 (w/v). The pH of the solution was adjusted to the optimal environment of the Alcalase 2.4 L (pH 9.0) using 0.1 N NaOH. The solution mixed with Alcalase 2.4 L with a dose of 1.85 % (v/w, based on the dry weight of seeds) was incubated in a shaker incubator at 100 rpm and 25 °C. During enzymatic hydrolysis process, a continuous shaking device was utilized to disperse the mixture, and the process were maintained at the aforementioned stable temperature and pH for a duration of 4 h. After completion of the EAEP, the mixed solution was centrifuged at 4500×*g* and 25 °C for 20 min to obtain three different liquid phases, namely oil, cream, and skim layers. The cream layer is a rich-oil fraction (oil-in-water emulsion), while the skim layer is an aqueous fraction that is rich in protein and sugar but lean in oil. In addition, there is an insoluble layer which is rich in fiber. The free oil was collected and transferred from the top oil phase.

### Extraction of phenolic compounds

The seed powders (20 g) of blue honeysuckle were mixed with 150 mL of methanol (80 %, v/v) by shaking for 2 h. The fully mixed solution was centrifuged at 5000×*g* and 25 °C for 10 min to obtain the supernatant. Addition of 50 mL of methanol (80 %, v/v) to the residues and repeat the above operation for 2 more times. All the collected supernatants were mixed and concentrated at 40 °C using a rotary evaporator to obtain concentrated crude extracts for subsequent analysis. The seed oil (2.0 g) was mixed with 5 mL of methanol (60 %, v/v) to obtain the phenolic extracts using a separating funnel. The volume of an aliquot methanol extract (1 mL) was diluted to 4.0 mL with distilled water. The phenolic extracts from seed oil were stored at 4 °C for further experiments.

### Determination of phenolic compounds in seeds and seed oil

#### Total phenolic content (TPC)

The TPC of samples was measured by Folin-Ciocalteu’s method reported by Waterhouse (2002) with few modifications. The 20 µL of diluted samples were darkly incubated with 10 µL of Folin-Ciocalteu’s reagent and 90 µL of distilled water in a 96 well-plate. After 5 min, 80 µL of Na_2_CO_3_ (75 g/L) was pipetted to provide alkaline environment. The mixture was cultured by a microplate reader (Epoch 2, BioTek, USA) and read the absorbance at 765 nm after 2 h. The TPC was calculated by gallic acid calibration curve. The standard curve was y = 6.41x + 0.0322, with R^2^ value of 0.99. The calculated TPC were expressed as milligram gallic acid equivalent per gram dry weight of samples (mg GAE/g DW).

#### Total flavonoid content (TFC)

The TFC of seeds and seed oil were determined according to the latest research of Dewanto, Wu, Adom, and Liu (2002) with minor modifications. The phenolic compounds (20 µL) from seed and seed oil were diluted with 120 µL of distilled water and 10 µL of 5 % NaNO_2_ in a 96 well-plate, respectively. After 6 min, addition of 20 µL of AlCl_3_·6H_2_O to mixed solution. The 40 µL of NaOH solution was added and reacted for 6 min. The mixture was cultured at 25 °C for 15 min and the absorbance at 510 nm was measured. Catechin was used as a standard to calculate the TFC of samples. The standard curve was y = 0.0208x + 0.0788, with R^2^ value of 0.99. The results were expressed as milligram catechin equivalent per gram dry weight of samples (mg CE/g DW).

#### Total anthocyanin content (TAC)

The pH difference method described by Giusti and Wrolstad (2001) was slightly modified to determine the TAC of seeds and seed oil. The samples were diluted with the freshly prepared sodium acetate buffer (0.4 M, pH 4.5) and potassium chloride buffer (0.025 M, pH 1.0). The 20 µL of diluted samples and prepared buffer solution was mixed, and the absorbance was accurately read at 510 nm and 700 nm using a microplate reader, respectively. The TAC was calculated using Eq. (1) and Eq. (2). The results were expressed as milligram C3G equivalent per gram of dry weight of samples (mg C3GE/g DW).

(1) $$A={\left(A_{510nm}\;-\;A_{700nm}\right)}_{pH1.0}-\;{\left(A_{510nm\;}-\;A_{700nm}\right)}_{pH4.5}$$

(2) $$C3G\;equivalent\;\left(\mathrm{mg}/g\;of\;dry\;weight\;of\;fruit\right)=\left(A\times MW\times DF\times 1000\right)/\left(\varepsilon \times 1\right)$$

where, A represents absorbance; MW represents the relative molecular weight of C3G, DF represents dilution factor; and ε represents molar absorptivity (26900).

#### Total proanthocyanidin content (TPAC)

The quantification of total proanthocyanidins in the samples was determined according to the method of DMAC recorded by Prior et al. (2010). The 70 µL of samples diluted with methanol (80 %, v/v) or standards was mixed with 210 µL of DMAC solution. The DMAC solution was freshly prepared with 0.1 % DMAC and acidified ethanol. The acidified ethanol was prepared with ethanol (91 %), deionized water and hydrochloric acid (36 %) at a volume ratio of 6:1:1. The absorbance at 640 nm measured using a microplate reader was brought into the standard calibration curve to calculate TPAC of the samples. The standard curve was y=24.303x+0.0151, with R^2^ value of 0.99. The results were expressed as milligram C3G equivalent per gram dry weight of samples (mg C3GE/g DW).

### Determination of antioxidant capacity in seeds and seed oil

#### DPPH assay

The DPPH assay used to determine the antioxidant capacity of samples was carried out the method of Brand-Williams, Cuvelier, and Berset (1995) with minor modifications. The 5 µL of gradient diluted samples or Trolox standards were darkly reacted with 195 µL of newly compounded DPPH solution (60 μM) in a 96-well plate for 2 h. The absorbance at 517 nm was readable using a microplate reader. The antioxidant capacity of samples was calculated by Trolox standard curve and expressed as micromole Trolox equivalent per gram of dry weight of samples (µmol TE/g DW).

#### ABTS assay

The analysis of ABTS was performed by the previous study of Pellegrini, Proteggente, Pannala, Yang and Rice-Evans (1999). To acquire the ABTS•^+^ solution, a freshly prepared mixture of ABTS solution (7 mM) and potassium persulfate solution (2.45 mM) was allowed to react fully in the dark at 25 °C for 12 h. The 10 µL of samples or Trolox standards were mixed with 190 µL of ABTS•^+^ solution at 25 °C in the dark. After 10 min, the absorbances of wells at 734 nm were immediately captured using a microplate reader. The standard curve was drawn by Trolox and calculated the antioxidant capacity. The ABTS radical scavenging ability was described by micromole Trolox equivalent per gram of dry weight of samples (µmol TE/g DW).

#### Ferric reducing antioxidant power (FRAP) assay

The determination of FRAP of blue honeysuckle seed extracts was referred to the previous research of Benzie and Strain (1996) with some modifications. The newly prepared TPTZ solution (10 mM TPTZ in 40 mM HCl) was mixed with FeCl_3_·6H_2_O (20 mM) and acetate buffer (300 mM, pH 3.6) at a volume ratio of 1:1:10 to obtain the FRAP solution. A 96 well-plate was filled with 150 µL of FRAP solution and incubated at 37 °C. After 30 min, the absorbance at 593 nm was measured. Addition of 10 µL of extracts or FeSO_4_·7H_2_O standards and 30 µL of deionized water to FRAP solution and read the absorbance of mixture at 593 nm and 37 °C in 30 min. The calculation result obtained by the standard curve (FeSO_4_·7H_2_O) was expressed as micromole Fe^2+^ equivalent per gram of dry weight of samples (µmol Fe^2+^E/g DW).

### Physicochemical analysis of seed oil

The El-Adawy and Taha’s method (2001) was used to determine the iodine value (method Cd 1-25), acid value (method Ca 3a-63), saponification value (method Cd 3-25), and peroxide value (method Cd 8-53) in the blue honeysuckle seed oil.

### Analysis of fatty acid composition in the seed oil by gas chromatography mass spectrometry (GC–MS)

The fatty acid composition in blue honeysuckle seed oil was analyzed using GC–MS. To increase the volatility of the seed oil, pre-column derivatization was used. In a 10 mL screw-cap glass tube, 30 µL of seed oil was dissolved in a mixed solution of *n*-hexane and benzene (1:1, v/v) and gently shaken. Then, 2 mL of 0.5 mol/L KOH in methanol was added, and the mixture was allowed to stand for 30 min. Subsequently, 5 mL of distilled water was added to separate the organic phase solution (*n*-hexane), causing it to rise to the upper layer of the tube. The top layer solution was diluted 20 times and analyzed by GC–MS on an Agilent 6890 GC system equipped with a 5973 N mass selective detector (Agilent Technologies Inc., Wilmington, DE, USA) using a DB-5 capillary column (60 m × 0.25 mm id, 0.25 µm film thickness, J&W Scientific, CA, USA). The GC injection port temperature was 250 °C, and helium was used as the carrier gas at a flow rate of 1.0 mL/min. The injection volume was 1.0 µL, and the injection split ratio was 1:10. The oven temperature was started at 180 °C and held for 5 min, and then ramped to 240 °C at 3 °C/min and held for 8 min. The total program time was 32 min. The ion source temperature was 230 °C. The MS detection was operated at 70 eV with a scan range of 50–550 *m*/*z*.

### Analysis of volatile components in the seeds and seed oil by headspace solid-phase microextraction (HS-SPME)-GC–MS

The volatile components of blue honeysuckle seeds and seed oil were extracted and concentrated using HS-SPME, and subsequently analyzed by GC–MS. The HS-SPME extraction procedure was as follows: 10 g of blue honeysuckle seeds or seed oil were transferred into a 10 mL screw-cap glass tube with a PTFE silicone septum and equilibrated in a 60 °C water bath for 5 min. The HS-SPME fiber (50/30 µm, divinylbenzene/carboxen/polydimethylsiloxane, Supelco, Bellefonte, PA, USA) was positioned in the headspace of the tube by inserting the HS-SPME needle into the PTFE silicone septum, and allowed to extract for 40 min at 60 °C. The fiber was pulled back into the needle and taken out of the tube following the extraction process. Subsequently, it was promptly introduced into the GC injection port to undergo thermal desorption. The fiber was exposed for 2 min at 250 °C injector temperature. GC–MS analysis was operated using an Agilent 6890 GC system with the 5973 N mass selective detector coupled with the DB-5 capillary column. A flow rate of 1.0 mL/min was maintained using helium as the carrier gas. The oven temperature was programmed as follows: started at 25 °C, raised to 80 °C and held for 2 min, and then increased to 230 °C at 6 °C/min and held for 13 min. The total program time was 35 min. The ion source temperature was 225 °C. The MS detection was conducted at 70 eV with a scan range of 50–500 amu.

### Analysis of relative odor active value (ROAV)

The contribution of different volatile components to the aroma of blue honeysuckle seeds and seed oil was evaluated using the ROAV. The ROAV of an individual volatile component was calculated by dividing its relative content by the corresponding sensory threshold (Zhang, et al., 2021). ROAV values ranging from 0 to 100 were used to indicate the intensity of different volatile components. Generally, volatile components with ROAV values greater than or equal to 1 were identified as key aroma components. Sensory thresholds were obtained from relevant literature.

### Analysis of tocopherols in seed oil by high-performance liquid chromatography with fluorescence detector (HPLC-FLD)

The tocopherol content of blue honeysuckle seed oil was determined using the method described by Li et al. (2011). Initially, 0.5 g of blue honeysuckle seed oil was mixed with 5 mL of hexane and filtered. The 20 µL of mixture was injected into a HPLC system (Shimadzu, Japan) equipped with a fluorescence detector (FLD). The excitation and emission wavelengths were set at 290 nm and 330 nm, respectively. A Phenomenex Luna Sil column (250 mm × 4.6 mm, i.d., and 5 μm particle size; Phenomenex, Inc., Torrance, CA) was used for separation. The mobile phase consisted of hexane and isopropanol (99/1, v/v), and the flow rate was set at 1.0 mL/min. To quantify the tocopherols present in blue honeysuckle seed oil, external standards for α-, β-, γ-, and δ-tocopherols were utilized.

### Analysis of phytosterols in seed oil

The analysis of phytosterols in blue honeysuckle seed oil was conducted with slight modifications to a previous method (Liu, Ou, Xiang, & Gregersen, 2020). The seed oil was saponified with ethanolic KOH and the unsaponifiable lipid layer was recovered and diluted to 50 mL with *n*-hexane. A 2 mL sample was pipetted to a 5 mL volumetric flask and dried using a water bath. Subsequently, the flask was supplemented with 0.5 mL of vanillin-glacial acetic acid (5 %) and 0.7 mL of perchloric acid, and then incubated at 70 °C for 30 min. Acetic acid was added until the volume reached 5 mL. The absorbance was recorded at 543 nm when the color of mixture changed.

### Identification of triglycerides in seed oil by high-performance liquid chromatography electrospray ionization mass spectroscopy (HPLC-ESI-MS^2^)

The triglycerides present in blue honeysuckle seed oil were analyzed using HPLC-ESI-MS^2^. The blue honeysuckle seed oil was dissolved in *n*-hexane and 10 µL of the solution (1 mg/mL) was injected into a HPLC system equipped with a C18 column. The mobile phases consisted of binary mobile phases A (acetonitrile and methanol solution at a volume ratio of 7:5) and B (2-propanol). The elution program initiated with 100 % A for 10 min, followed by a linear gradient to 100 % B at 30 min, and finally changed to 100 % A in 1 min and held for 9 min. The absorbance of triglycerides was detected at 215 nm. Acquisition of the full scan mass spectra was performed using a collision energy of 10 eV under positive ion mode, from an *m*/*z* range of 100 to 1000. For positive ion ESI, the capillary voltage was set to −4.5 KV, and a dry 99 % N_2_ gas was used at 180 °C. Compass data analysis version 4.0 (Bruker Daltronics, Bremen, Germany) was utilized for processing the acquired spectra.

### Proximate analysis

We employed AOAC standard methods to analyze the fat, ash, moisture, fiber, and protein content of the seeds. The crude fat content was determined using the petroleum ether extraction method (AOAC 930.09). Ash content was obtained through sample incineration in a muffle furnace at 550 °C (AOAC 930.05). Moisture content was determined by hot air drying the samples (AOAC 990.19). Total fiber content was assessed using the enzymatic gravimetric method (AOAC 985.29). The crude protein content of the samples was evaluated using the Kjeldahl method (AOAC 978.04). Carbohydrate content was calculated using the difference method.

### Statistical analysis

The experimental data were analyzed using a Student's *t*-test with SPSS software (v20.0, IBM SPSS, Chicago, IL, USA), and the results were expressed as means and standard deviations of triplicates. Differences were determined to be statistically significant when p < 0.05. The data was analyzed statistically using Microsoft Excel (v2019, Microsoft, Redmond, WA, USA).

## Results and discussion

### Quantitation of phenolic compounds in blue honeysuckle seeds and seed oil

**Fig. 1:**
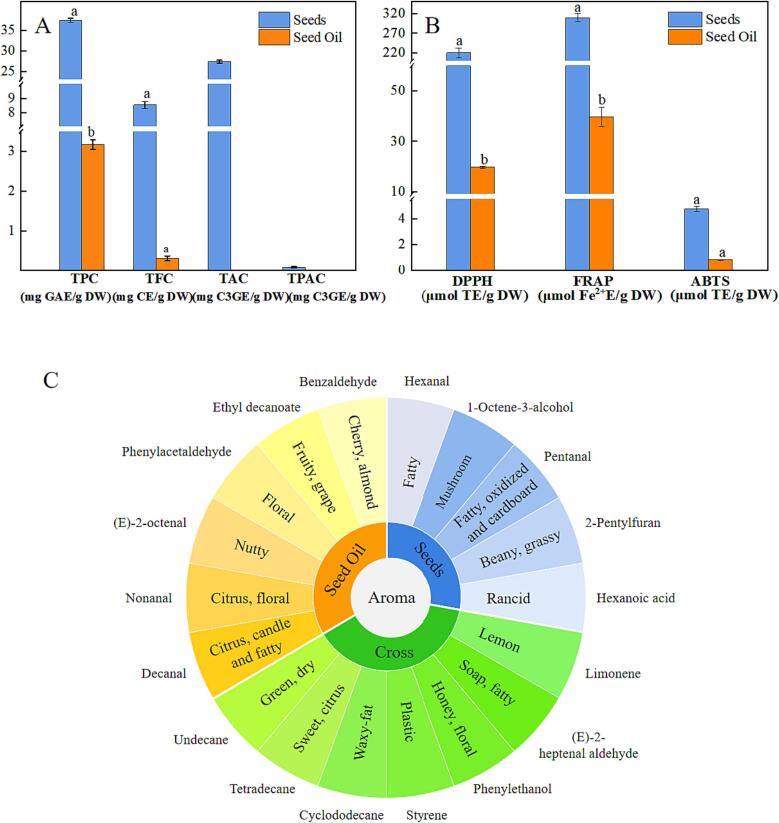
Quantitation of phenolic compounds (A), antioxidant capacity (B), and aroma characteristics of volatile components (C) of blue honeysuckle seeds and seed oil. (For interpretation of the references to color in this figure legend, the reader is referred to the web version of this article.)

The results of total phenolic content (TPC), total anthocyanin content (TAC), total flavonoid content (TFC), and total proanthocyanidin content (TPAC) of blue honeysuckle seeds and seed oil were illustrated in Fig. 1A and Table S1. The TPC and TFC values of blue honeysuckle seeds and seed oil were measured. The TPC value of blue honeysuckle seeds was 37.40 ± 0.52 mg GAE/g DW, more than double that of blueberry seeds (15.8 ± 0.63 mg GAE/g DW) (Parry, et al., 2006). In contrast, the TPC and TFC values of blue honeysuckle seed oil were 3.16 ± 0.11 mg GAE/g DW and 0.30 ± 0.06 mg CE/g DW, respectively. The TPC value of blue honeysuckle seed oil was slightly higher than that of other berries seed oil such as blueberry seed oil (1–1.73 mg GAE/g DW), red raspberry seed oil (0.16–2 mg GAE/g DW), and marion berry seed oil (0.1–1.49 mg GAE/g DW) (Parry, et al., 2005). The reason for TPC and TFC values of blue honeysuckle seed oil being lower than that of seeds can be attributed to the fact that polyphenols are hydrophilic substances and have low solubility in seed oil. In addition, the determination of TPC has some drawbacks, such as the possibility of false positives caused by the reaction between Folin-Ciocalteu’s reagent and other non-phenolic compounds. Therefore, interpreting TPC as antioxidant’s reducing capacity may be more accurate (Huang, Ou, & Prior, 2005).

The majority of research on anthocyanins and proanthocyanidins has focused on fruits, with limited research on fruit seeds. In this study, the TAC value of blue honeysuckle seeds (27.25 ± 0.03 mg C3GE/g DW) was higher than that of black mulberry (0.57–1.88 mg C3GE/g DW) (Babbar, Oberoi, Uppal, & Patil, 2011), while the TPAC value of blue honeysuckle seeds was quite low (0.073 ± 0.02 mg C3GE/g DW) and rarely reported in the literature. In addition, the TAC and TPAC values of blue honeysuckle seed oil were negligible, likely due to the water-solubility of anthocyanins and proanthocyanidins and their poor solubility in seed oil. Therefore, blue honeysuckle seeds and seed oil rich in polyphenols can be considered natural sources of nutrition for human health.

### Antioxidant capacity of blue honeysuckle seeds and seed oil

The stability of seed oil is a critical factor in determining its long-term storage potential. To assess the radical-scavenging capacity and oxidation stability of blue honeysuckle seed oil, we employed the determination of antioxidant capacity in our study. The DPPH, ABTS and FRAP assays with different radical quenching mechanisms were used to determine the antioxidant capacity of blue honeysuckle seeds and seed oil, which made our study more comprehensive and authoritative. As shown in Fig. 1B, the DPPH, ABTS and FRAP values of blue honeysuckle seeds were 220.44 ± 12.14 μmol TE/g DW, 4.75 ± 0.20 μmol TE/g DW, and 308.96 ± 10.63 μmol Fe^2+^/g DW, respectively. Remarkably, the DPPH and FRAP values of blue honeysuckle seeds were almost four times higher than those of ‘Black Pearl’ grape seeds (65.11 ± 1.14 μmol TE/g DW and 84.47 ± 1.08 μmol Fe^2+^/g DW) (Xu, Zhang, Cao, & Lu, 2010). The findings of this study suggest that blue honeysuckle seeds are a potential source of antioxidants that have been overlooked in the past. These results indicate that the seeds could be utilized in further food processing. Additionally, the DPPH, ABTS and FRAP values of blue honeysuckle seed oil were 19.67 ± 0.32 µmol TE/g DW, 0.84 ± 0.03 μmol TE/g DW, and 39.75 ± 3.80 μmol Fe^2+^E/g DW, respectively. A previous study conducted by Zheng et al. (2017) showed that the FRAP and DPPH values of sea-buckthorn seed oil were 1.68–2.17 µmol TE/g and 21.4–28.1 µmol TE/g, respectively, which were lower than those of blue honeysuckle seed oil. Several previous studies have indicated that berries rich in polyphenols generally exhibit higher antioxidant capacity, possibly due to the hydrogen atom-donating ability of phenolic compounds with hydroxyl groups (–OH) that scavenge free radicals (Mustafa, et al., 2022). Therefore, the lower antioxidant capacity of seed oil is due to the absence of hydrophilic polyphenols. Furthermore, as shown in Fig. 1B, the ABTS values of blue honeysuckle seeds and seed oil were found to be lower than those of DPPH and FRAP. This difference in antioxidant capacity may be attributed to the distinct reaction characteristics and mechanisms of each assay, as reported by Huang, Ou and Prior (2005). Given the observed antioxidant capacity, blue honeysuckle seeds and seed oil could potentially serve as valuable natural sources of phenolic compounds in the human diet.

### Analysis of physicochemical properties of blue honeysuckle seed oil

To determine the physicochemical properties of the blue honeysuckle seed oil, four common indicators were measured, including acid value, saponification value, iodine value, and peroxide value. The acid value is a typical indicator of the quantity of free fatty acids in oil, and is employed to reflect the quality and freshness of the oil. Seed oil with lower acid value is generally more resistant to storage and more suitable for human consumption (Konuskan, Arslan, & Oksuz, 2019). Compared with acid values of chokeberry seed oil (8.9 mg KOH/g) and raspberry seed oil (10.5 mg KOH/g), blue honeysuckle seed oil with lower acid value (5.14 ± 0.35 mg KOH/g oil) was a more reliable choice for seed oil (Mazurek, Ryszko, Kostrzewa, Chmiel, & Kondracka, 2022).

Iodine value is an accurate indicator of oil unsaturation and is positively correlated with oil unsaturation. Generally, oil with higher iodine value contains more unsaturated bonds, which are more beneficial to human health. The iodine value of blue honeysuckle seed oil was 126.9 ± 5.73 I_2_/100 g oil, which was higher than that of olive oil, peanut oil, and sunflower seed oil (ranging from 80.03 to 111.19 I_2_/100 g oil) (Konuskan, Arslan, & Oksuz, 2019). Blue honeysuckle seed oil with higher iodine value is a good source of UFA.

The saponification value is used to reflect the relative molecular mass of the oil and has an inverse relationship. As shown in Table 1, the saponification value of blue honeysuckle seed oil was 180.92 ± 11.07 mg KOH/g oil, indicating the high content of linoleic acid (Kapoor, Gandhi, Tyagi, Kaur, & Mahajan, 2020).

**Table 1:** Analysis of physicochemical properties, tocopherols, and phytosterols of blue honeysuckle seed oil.

	Value
Physicochemical properties	
Acid value (mg KOH/g oil)	5.14 ± 0.35
Iodine value (g I_2_/100 g oil)	126.9 ± 5.73
Saponification value (mg KOH/g oil)	180.92 ± 11.07
Peroxide value (mmol/kg oil)	6.55 ± 0.29
Tocopherol and phytosterols	
α-tocopherol (mg/kg oil)	23.08 ± 1.23
β-tocopherol (mg/kg oil)	0.54 ± 0.01
γ-tocopherol (mg/kg oil)	205.47 ± 10.81
δ-tocopherol (mg/kg oil)	2.31 ± 0.15
Phytosterols (mg/kg oil)	130 ± 2.57

The peroxide value is an important index to determine the degree of oil oxidation. A higher peroxide value commonly indicates more oxidation products present in the seed oil, which may lead to diarrhea. The peroxide value of blue honeysuckle seed oil was 6.55 ± 0.29 mmol/kg oil, which conformed to the international edible vegetable oil standard that the peroxide value shall not exceed 7.5 mmol/kg (Hromiš et al., 2022).

The blue honeysuckle seed oil meets the oil edible standard, and this new functional seed oil, with higher iodine value and lower peroxide value, adds more choice to our table.

Additionally, proximate analysis of blue honeysuckle seeds was determined and summarized in Table S2.

### Fatty acid composition of blue honeysuckle seed oil

The fatty acid composition and relative content of blue honeysuckle seed oil were analyzed by GC–MS and are shown in Table 2. Eight types of fatty acids were detected in blue honeysuckle seed oil, with linoleic acid and elaidic acid being the most abundant (71.24 ± 1.64 % and 20.74 ± 1.47 %, respectively). The contents of the other five fatty acids were extremely low, including gondoic acid, 14-methylpentadecanoic acid, oleic acid, 15-methyl heptadecanoic acid, myristic acid and palmitic acid. Fatty acids can be classified into saturated fatty acids (SFA) and UFA. Interestingly, the majority of the fatty acids in blue honeysuckle seed oil were UFA, which was consistent with the iodine value result. Previous studies have demonstrated that UFA can regulate blood lipids and prevent cardiovascular diseases (Göktürk Baydar, Özkan, & Yaşar, 2007). The content ratio of PUFA to monounsaturated fatty acids (MUFA) was approximately 3:1. Additionally, the ratio of PUFA to SFA in blue honeysuckle seed oil was up to 12.38, which greatly exceeds the recommended ratio of 0.45 set by the British Department of Health (De Alba et al., 2019). The increased intake of PUFA in daily life can effectively decrease the risk of cardiovascular and coronary heart diseases (de Alba, Pérez-Andrés, Harrison, Brunton, Burgess, & Tiwari, 2019). Pieszka et al. (2015) found that the ratio of PUFA to SFA of raspberry and blackcurrant seed oil were around 8.18 and 6.63, respectively. Although the UFA in above berry seed oil accounts for the majority of fatty acids, the linoleic acid content in raspberry seed oil (49.01 %), strawberry seed oil (45.45 %), and blackcurrant seed oil (38.64 %) was lower than that of blue honeysuckle seed oil. The increased concentration of linoleic acid in the ω-6 series in blue honeysuckle seed oil provides a reliable source for humans to effectively prevent coronary heart disease, colon cancer, and skin cancer (Pieszka et al., 2015). The blue honeysuckle seed oil also contained a small quantity of oleic acid (1.86 ± 0.08 %), a type of MUFA that can regulate cholesterol levels and prevent arteriosclerosis (Pieszka et al., 2015). Therefore, the high content of UFA and low levels of SFA in blue honeysuckle seed oil make it an excellent substitute for edible oil.

**Table 2:** The GC–MS analysis of fatty acids and LC-MS analysis of triglycerides of blue honeysuckle seed oil.

Peak	RT (min)	Compounds	MW	[M + Na]^+^	MS^2^	Formula	Content (%)	Type
Fatty acids								
1	18.04	14-methylpentadecanoic acid	256.42	–	–	C_16_H_32_O_2_	2.23 ± 0.08	SFA
2	19.42	Gondoic acid	310.51			C_20_H_38_O_2_	0.41 ± 0.02	MUFA (ω-9)
3	23.02	Linoleic acid	280.44	–	–	C_18_H_32_O_2_	71.24 ± 1.64	PUFA (ω-6)
4	23.14	Elaidic acid	282.46	–	–	C_18_H_34_O_2_	20.74 ± 1.47	MUFA (ω-9)
5	23.28	Oleic acid	282.46	–	–	C_18_H_34_O_2_	1.86 ± 0.08	MUFA (ω-9)
6	23.81	15-methylheptadecanoic acid	284.48	–	–	C_20_H_40_O_2_	1.83 ± 0.17	SFA
7	24.60	Myristic acid	228.37			C_14_H_28_O_2_	0.95 ± 0.01	SFA
8	26.17	Palmitic acid	256.42			C_16_H_32_O_2_	0.74 ± 0.02	SFA
Triglycerides								
1	22.22	LLLn	876.71	899.71	597.49, 619.47	C_57_H_96_O_6_	–	–
2	22.58	LLL	878.72	901.72	621.45	C_57_H_98_O_6_	–	–
3	22.97	OLL	880.74	903.74	621.49, 623.50	C_57_H_100_O_6_	–	–
4	22.98	LLP	854.72	877.72	597.49, 621.49	C_55_H_98_O_6_	–	–
5	23.31	OOL	882.76	905.76	623.50, 625.51	C_57_H_102_O_6_	–	–
6	23.66	OOO	884.77	907.77	625.51	C_57_H_104_O_6_	–	–

### Profiling of volatile components in blue honeysuckle seeds and seed oil

The volatile components of blue honeysuckle seeds and seed oil were subjected to HS-SPME-GC–MS analysis. As shown in Table 3, 34 and 37 volatile components were tentatively identified in seeds and seed oil, respectively. In blue honeysuckle seeds, the 34 volatile components were classified into five categories based on their functional groups, including 12 hydrocarbons, 6 aldehydes, 7 alcohols, 4 esters, 2 carboxylic acids, 1 ketone, and 2 other compounds. On the other hand, 37 volatile components were tentatively identified in blue honeysuckle seed oil, including 20 hydrocarbons, 8 aldehydes, 2 alcohols, 4 esters, and 3 carboxylic acids. It was obvious that aldehydes were the major volatile components in both blue honeysuckle seeds and seed oil, accounting for 18.94 % of seeds and 24.37 % of seed oil. The increase in aldehydes in seed oil may be attributed to the degradation of UFA. This was consistent with previous research by Bederska-Łojewska, who also found that aldehydes were the main volatile components in raspberry and blueberry seeds (Bederska-Łojewska et al., 2021). The (E)-2-heptene aldehyde had the highest content among the aldehydes of seed oil, indicating that it may contribute to the aroma. Hexanal had the highest content among the aldehydes in seeds, which may be due to the degradation of linoleic acid (Bail, Stuebiger, Krist, Unterweger, & Buchbauer, 2008). Additionally, alcohols were the second most abundant volatile components in seeds and seed oil. The content of alcohols in seed oil (20.95 %) was higher than that in seeds (17.72 %), possibly due to the lipid degradation in seed oil. It is noteworthy that seeds and seed oil were found to have the most diverse hydrocarbons, which were produced through biosynthesis pathways. However, the hydrocarbons with higher sensory thresholds were found to contribute less to the overall aroma.

**Table 3:** The HS-SPME-GC–MS analysis of the volatile components of blue honeysuckle seeds and oil.

Category	Peak	Compounds	Sensory Threshold (μg/kg)	Formula	Content (%)	
					Seed oil	Seeds
Hydrocarbons	1	Styrene	730	C_8_H_8_	0.52 ± 0.04	4.00 ± 0.13
	2	1-methyl-3-(1-methylethyl)-benzene	NF	C_10_H_14_	1.28 ± 0.09	–
	3	Cyclododecane	2040	C_12_H_24_	1.96 ± 0.11	1.18 ± 0.23
	4	Cyclooctane	NF	C_8_H_16_	1.03 ± 0.09	–
	5	1-methyl-4-(1-methylvinyl) benzene	NF	C_10_H_12_	0.43 ± 0.06	–
	6	(1S)-3,7,7-trimethylbicyclohept-3-ene	NF	C_10_H_16_	1.69 ± 0.41	–
	7	1-methyl-4-(1-methylethyl)-1,4-cyclohexadiene	NF	C_10_H_16_	0.93 ± 0.02	–
	8	3,7-dimethyldecane	NF	C_12_H_26_	0.63 ± 0.02	–
	9	Tridecane	NF	C_13_H_28_	0.59 ± 0.01	0.94 ± 0.13
	10	Octacosane	NF	C_28_H_58_	0.78 ± 0.09	–
	11	Pentadecane	NF	C_25_H_52_	0.13 ± 0.02	–
	12	Tetradecane	1000	C_14_H_30_	0.60 ± 0.04	0.64 ± 0.02
	13	Hexacosane	NF	C_26_H_54_	0.37 ± 0.03	–
	14	Pentadecane	NF	C_15_H_32_	0.47 ± 0.02	–
	15	Eicosane	NF	C_20_H_42_	0.53 ± 0.03	–
	16	*N*-hexadecane	NF	C_16_H_34_	0.18 ± 0.07	–
	17	Limonene	10	C_10_H_16_	3.71 ± 0.49	2.97 ± 0.78
	18	Decane	NF	C_10_H_22_	–	0.26 ± 0.07
	19	Pentamethylheptane	NF	C_12_H_26_	–	1.58 ± 0.46
	20	Undecane	2140	C_11_H_24_	–	0.47 ± 0.08
	21	Hexamethylcyclotrisiloxane	NF	C_6_H_18_O_3_Si_3_	1.05 ± 0.36	–
	22	Decamethylcyclopentasiloxane	NF	C_10_H_30_O_5_Si_5_	0.78 ± 0.53	2.02 ± 0.14
	23	Dodecyl cyclohexasiloxane	NF	C_12_H_36_O_6_Si_6_	0.23 ± 0.04	–
	24	3,3-dimethyl-1,2-epoxybutane	NF	C_6_H_12_O	–	0.73 ± 0.32
	25	（Z)-2-dodecene	NF	C_12_H_24_	–	0.68 ± 0.11
	26	（E)-2-tetradecene	NF	C_14_H_28_	–	1.73 ± 0.28
Aldehydes	27	（Z)-2-heptanaldehyde	NF	C_7_H_12_O	0.50 ± 0.08	–
	28	Benzaldehyde	350	C_7_H_6_O	2.82 ± 0.21	–
	29	Phenylacetaldehyde	4	C_8_H_8_O	2.27 ± 0.42	–
	30	(E)-2-Octenal	3	C_8_H_14_O	3.02 ± 0.38	–
	31	Nonanal	1	C_9_H_18_O	5.28 ± 0.51	–
	32	(E)-Nonenal	NF	C_9_H_16_O	1.30 ± 0.29	–
	33	Decanal	0.1	C_10_H_20_O	1.45 ± 0.71	–
	34	2-Dodecenal	NF	C_12_H_22_O	–	3.23 ± 0.58
	35	3-methylbutyraldehyde	NF	C_5_H_10_O	–	0.27 ± 0.04
	36	Pentanal	12	C_5_H_10_O	–	0.25 ± 0.07
	37	Hexanal	4.5	C_6_H_12_O	–	12.85 ± 3.14
	38	(Z)-2-Dodecene	NF	C_6_H_10_O	–	0.68 ± 0.03
	39	(E)-2-heptene aldehyde	13	C_7_H_12_O	7.63 ± 0.84	1.66 ± 0.53
Alcohols	40	Menthol	NF	C_10_H_20_O	0.52 ± 0.03	0.18 ± 0.05
	41	Phenylethanol	750	C_8_H_10_O	20.43 ± 3.16	2.84 ± 0.49
	42	3,7-dimethyl-1,6-octadiene-3-ol	NF	C_10_H_18_O	–	4.75 ± 0.08
	43	1-octene-3-alcohol	1	C_8_H_16_O	–	3.08 ± 0.39
	44	Eucalyptol	NF	C_10_H_18_O	–	3.44 ± 0.52
	45	3,7-dimethyl-1,6-octadiene-3-ol	NF	C_10_H_18_O_2_	–	2.59 ± 0.64
	46	Terpinen-4ol	NF	C_10_H_18_O	–	0.84 ± 0.02
Esters	47	Ethyl acetate	NF	C_4_H_8_O_2_	0.10 ± 0.04	1.59 ± 0.36
	48	Ethyl decanoate	5	C_12_H_24_O_2_	0.14 ± 0.05	–
	49	9-hexadecenoic acid ethylester	NF	C_18_H_34_O_2_	1.91 ± 0.13	–
	50	Ethyl palmitate	NF	C_18_H_36_O_2_	0.74 ± 0.04	–
	51	3-methyl-1butanolacetate	NF	C_7_H_14_O_2_	–	0.88 ± 0.41
	52	Methyl caproate	NF	C_7_H_14_O_2_	–	0.26 ± 0.05
	53	Nonyl acetate	NF	C_11_H_22_O_2_	–	1.26 ± 0.09
	54	Diethyl phthalate	NF	C_12_H_14_O_4_	–	2.91 ± 0.86
Carboxylic acids	55	*n*-hexadecanoic acid	NF	C_16_H_32_O_2_	0.75 ± 0.26	–
	56	(Z,Z)-9,12-octadecadienoic acid	NF	C_18_H_32_O_2_	5.32 ± 0.31	–
	57	(E)-9-octadecenoic acid	NF	C_18_H_34_O_2_	1.54 ± 0.27	–
	58	Hexanoic acid	3000	C_6_H_12_O_2_	–	3.58 ± 0.23
	59	Heptanoicacid	NF	C_7_H_14_O_2_	–	2.93 ± 0.52
Ketones	60	5-methyl-2-hexanone	NF	C_7_H_14_O	–	0.34 ± 0.05
Othercompounds	61	2-pentylfuran	6	C_9_H_14_O	–	2.01 ± 0.42
	62	Butyl hydroxytoluene	NF	C_15_H_24_O	–	–

Note: NF indicates that the sensory threshold of the substance is not found.

The ROAV has a crucial function in determining the aroma characteristics of blue honeysuckle seeds and seed oil. Volatile components with an ROAV of ≥1 are considered key aroma components, while those with an ROAV between 0.1 and <1 are potential aroma components that can modify the overall aroma (Zhang et al., 2021). Table 4 and Fig. 1C illustrate the ROAVs and aroma characteristics of volatile components in blue honeysuckle seeds and seed oil. The seeds and seed oil contained 4 and 6 compounds with an ROAV of ≥1, respectively. Key aroma components in the seed oil included decanal, which contributed to a citrus, candle, and fatty aroma, and nonanal, which had a distinct citrus and fresh floral scent. In the seeds, 1-octene-3-ol and hexanal were considered vital aroma components, contributing to mushroom and fatty aroma, respectively. Both seeds and seed oil contained limonene, which produced a lemon aroma, and (E)-2-heptene aldehyde, which produced a soap and fatty aroma. The compound limonene, which is a major contributor to flavor, has been detected in various berries and their seed oil, such as chokeberry, raspberry, and blackcurrant (Mildner-Szkudlarz, Różańska, Gaca, & Jeleń, 2021). Sevindik et al. (2021) found that nonanal was the most recognizable smell in grape seed oil. The aldehydes, especially hexanal, were the primary aroma contributors in strawberry and blackcurrant seed oil, similar to blue honeysuckle seed oil (Mildner-Szkudlarz, Różańska, Gaca, & Jeleń, 2021).

**Table 4:** ROAVs and aroma characteristics of volatile components of blue honeysuckle seeds and seed oil.

Compounds	Aroma characteristics	Sensory threshold (μg/kg)	ROAVs	
			Seed oil	Seeds
Styrene	Plastic aroma	730	< 0.1	0.18
Cyclododecane	Waxy-fat aroma	2040	< 0.1	< 0.1
Tetradecane	Sweet and citrus aroma	1000	< 0.1	< 0.1
Limonene	Lemon aroma	10	2.55	9.64
Undecane	Green and dry aroma	2140	< 0.1	< 0.1
Benzaldehyde	Cherry and almond aroma	350	< 0.1	–
Phenylacetaldehyde	Floral aroma	4	3.91	–
(E)-2-octenal	Nutty aroma	3	6.94	–
Nonanal	Citrus and fresh floral aroma	1	36.41	–
Decanal	Citrus, candle and fatty aroma	0.1	100	–
Pentanal	Fatty, oxidized and cardboard aroma	12	–	0.68
Hexanal	Fatty aroma	4.5	–	92.7
(E)-2-heptenal aldehyde	Soap and fatty aroma	13	4.04	4.14
Phenylethanol	Honey and floral aroma	750	0.22	0.12
1-octene-3-alcohol	Mushroom aroma	1	–	100
Ethyl decanoate	Fruity and grape aroma	5	0.19	–
Hexanoic acid	Rancid aroma	3000	–	< 0.1
2-pentylfuran	Beany and grassy aroma	6	–	0.11

### Analysis of tocopherols and phytosterols in blue honeysuckle seed oil

Tocopherols, which are fat-soluble vitamins, are valuable natural antioxidants. The contents of four tocopherol homologues (α, β, γ, and δ) in blue honeysuckle seed oil were analyzed for the first time using HPLC-PDA and are shown in Table 1. The total tocopherol content of blue honeysuckle seed oil was 231.49 ± 11.12 mg/kg oil, which was higher than that of kiwifruit (38.1 ± 2.7 mg/kg oil) and blueberry (102.5 ± 2.4) (Van Hoed et al., 2011). The predominant tocopherol in blue honeysuckle seed oil was γ-tocopherol, which constituted 88 % of the total tocopherols with a content of 205.47 ± 10.81 mg/kg oil. This amount was higher than that of kiwifruit seed oil (38.1 ± 2.7 mg/kg oil), cranberry seed oil (62.8 ± 3.7 mg/kg oil), and blueberry seed oil (53.9 ± 1.0 mg/kg oil) (Van Hoed et al., 2011). This finding was consistent with the tocopherol contents of raspberry, blackcurrant, strawberry, and chokeberry seed oil, which were also dominated by γ-tocopherol at around 70 % (Mildner-Szkudlarz, Różańska, Siger, Kowalczewski, & Rudzińska, 2019). According to a previous study by Hwang and Winkler-Moser (2017), γ-tocopherol had the strongest antioxidant capacity among the four tocopherols. Blue honeysuckle seed oil also contained a slightly higher content of α-tocopherol (23.08 ± 1.23 mg/kg) than black mulberry (8.05 ± 0.35 mg/kg), and white mulberry (6.60 ± 0.25 mg/kg) seed oil (Yılmaz & Durmaz, 2015). The presence of α-tocopherol was associated with antioxidant capacity and anti-inflammatory activity (Gustinelli, Eliasson, Svelander, Alminger, & Ahrné, 2018). Therefore, the blue honeysuckle seed oil showed potent antioxidant capacity because of its relatively high contents of γ-tocopherol and α-tocopherol. Furthermore, the detection of a substantial amount of tocopherols and phenolic compounds in the seed oil provides additional evidence that despite containing up to 93.84 % UFA, the seed oil still exhibited a robust antioxidant capacity. In addition, the total phytosterol content of blue honeysuckle seed oil was determined to be 130 ± 2.57 mg/kg oil. The intake of phytosterols from seed oil may be a potential way to decrease the risk of cardiovascular disease (Pieszka et al., 2015). This study analyzed the total phytosterol content of blue honeysuckle seed oil for the first time. However, further research is needed to determine the specific types of phytosterols present and their respective concentrations.

### Identification of triglycerides in blue honeysuckle seed oil

**Fig. 2:**
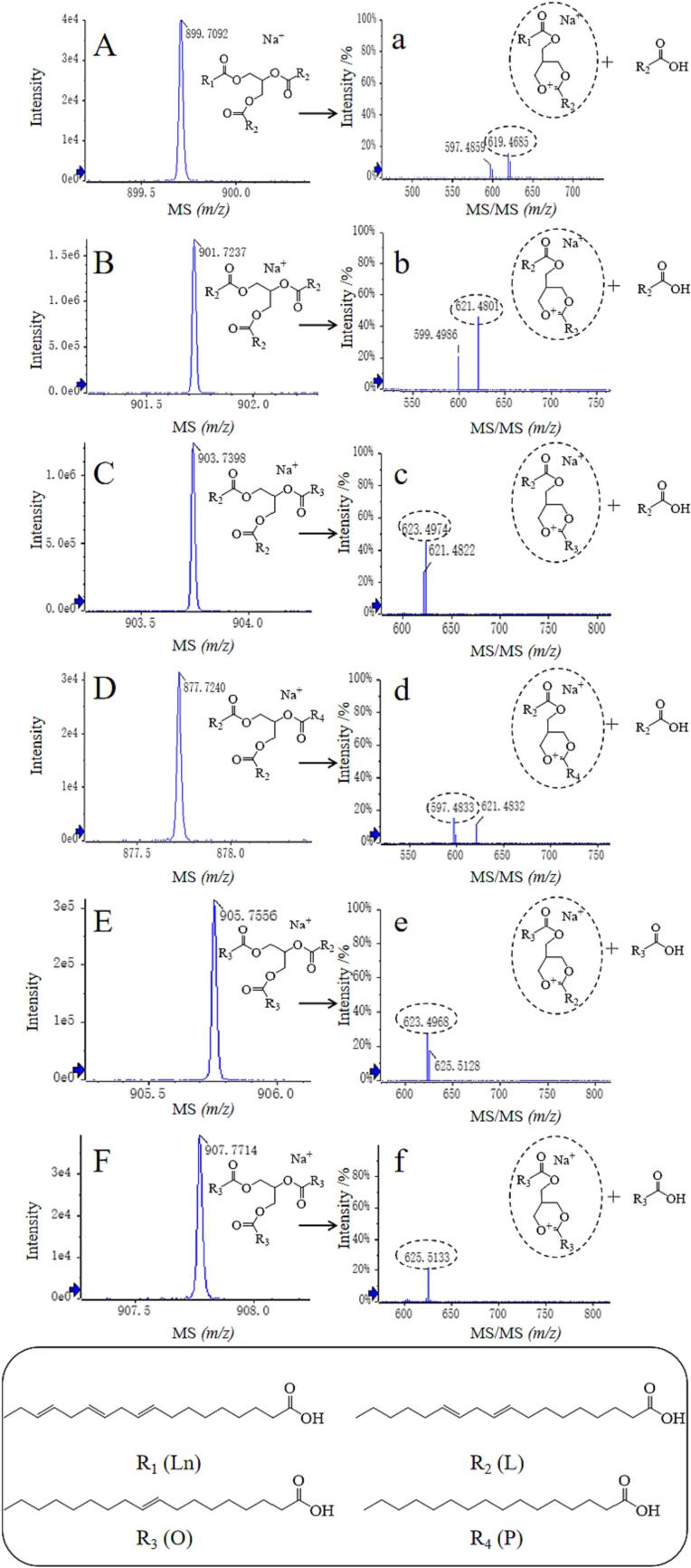
The primary mass spectra of triglycerides of blue honeysuckle seed oil of (A) LLLn, (B) LLL, (C) OLL, (D) LLP, (E) OOL, and (F) OOO. The secondary mass spectra of triglycerides of (a) LLLn, (b) LLL, (c) OLL, (d) LLP, (e) OOL, and (f) OOO. (For interpretation of the references to color in this figure legend, the reader is referred to the web version of this article.)

Triglycerides are one of the important factors in determining the physicochemical properties of seed oil. The triglycerides are complex mixtures that consist of one mole of glycerol and three moles of fatty acids, which are degraded to provide energy for humans (Yang et al., 2019). In this study, the triglycerides present in blue honeysuckle seed oil were analyzed using HPLC-ESI-MS^2^, which is a more sensitive and precise method for determining their compositions. The MS spectra of triglycerides in blue honeysuckle seed oil were shown in Fig. 2, and the corresponding data were summarized in Table 2. Six triglycerides were detected in blue honeysuckle seed oil, consisting of four types of fatty acids, namely linoleic acid (L, 280 Da), linolenic acid (Ln, 278 Da), palmitic acid (P, 256 Da), and oleic acid (O, 282 Da). Peak 1 showed a quasi-molecular ion at *m*/*z* 899.7 [M + Na]^+^. The fragment ions at *m*/*z* 619.4 (LLn) corresponded to the loss of L, and peak 1 was tentatively identified as LLLn. Peaks 2 and 4, with fragment ions at *m*/*z* 621.4 (LL) and *m*/*z* 597.4 (LP) were attributed to the loss of L. Therefore, peaks *P*2 and *P*4 were tentatively identified as LLL and LLP, respectively. The peaks 3 ([M + Na]^+^, *m*/*z* 903.7) and 5 ([M + Na]^+^, *m*/*z* 905.7) were tentatively characterized as OLL and OOL, exhibiting the same fragment ion at *m*/*z* 623.4 (OL). Peak 6 showed a quasi-molecular ion at *m*/*z* 907.7 [M + Na]^+^ and a fragment ion at *m*/*z* 625.4 [M + Na-282]^+^ (loss of O), which was tentatively assigned as OOO. From Fig. 2, it is evident that the highest relative abundances of LLL were detected among the six triglycerides, thereby confirming that linoleic acid was the predominant fatty acid present in the seed oil. The LLLn, LLL, and OLL were also detected in blackberry, black raspberry, and blueberry seed oil (Li, Wang, & Shahidi, 2016). A previous study had demonstrated that OLL, OOL, and OOO increased the oxidative stability of seed oil (Li, Wang, & Shahidi, 2016). Moreover, a significant quantity of phenolic compounds and tocopherols with strong antioxidant capacity were detected in blue honeysuckle seed oil, which can improve the oxidative stability of seed oil. Therefore, the blue honeysuckle seed oil was more resistant to storage because of these oxidation stabilizers.

## Conclusions

This study investigated the nutritional profile of blue honeysuckle seeds and seed oil for the first time. A total of 34 and 37 volatile components were tentatively identified in the seeds and seed oil, respectively, with aldehydes and alcohols being the major volatile components. In addition, 8 fatty acids, 4 tocopherol homologues (α, β, γ, and δ), and 6 triglycerides were found in the seed oil. Among them, linoleic acid was the most predominant fatty acid, which had a content of 71.24 ± 1.64 %. The relative high contents of γ-tocopherol and α-tocopherol, and the presence of OLL, OOL, and OOO of triglycerides contributed to the seed oil’s stronger antioxidative capacity and oxidative stability. Overall, this study demonstrated that blue honeysuckle seed oil obtained using the aqueous enzymatic method was a reliable and healthy option, with a delightful flavor, superior PUFA content, and potent antioxidant capacity.

## CRediT authorship contribution statement

**Juan Sun:** Writing – original draft. **Dalong Li:** Formal analysis. **Wenjing Huyan:** Methodology. **Xiaoqi Hong:** Data curation. **Shuman He:** Investigation. **Junwei Huo:** Resources. **Lianzhou Jiang:** Validation. **Yan Zhang:** Conceptualization, Funding acquisition, Validation, Writing – review & editing.

## Declaration of competing interest

The authors declare that they have no known competing financial interests or personal relationships that could have appeared to influence the work reported in this paper.

## Data Availability

Data will be made available on request.
