# Supplementary material for: Blue honeysuckle seeds and seed oil: Composition, physicochemical properties, fatty acid profile, volatile components, and antioxidant capacity
Source: Food Chem X. 2024 Feb 7;21:101176. doi: 10.1016/j.fochx.2024.101176 (PMC10877549; doi:10.1016/j.fochx.2024.101176)
Supplement: Supplementary data 1 [file mmc1.docx]

**Supplementary Material**

**
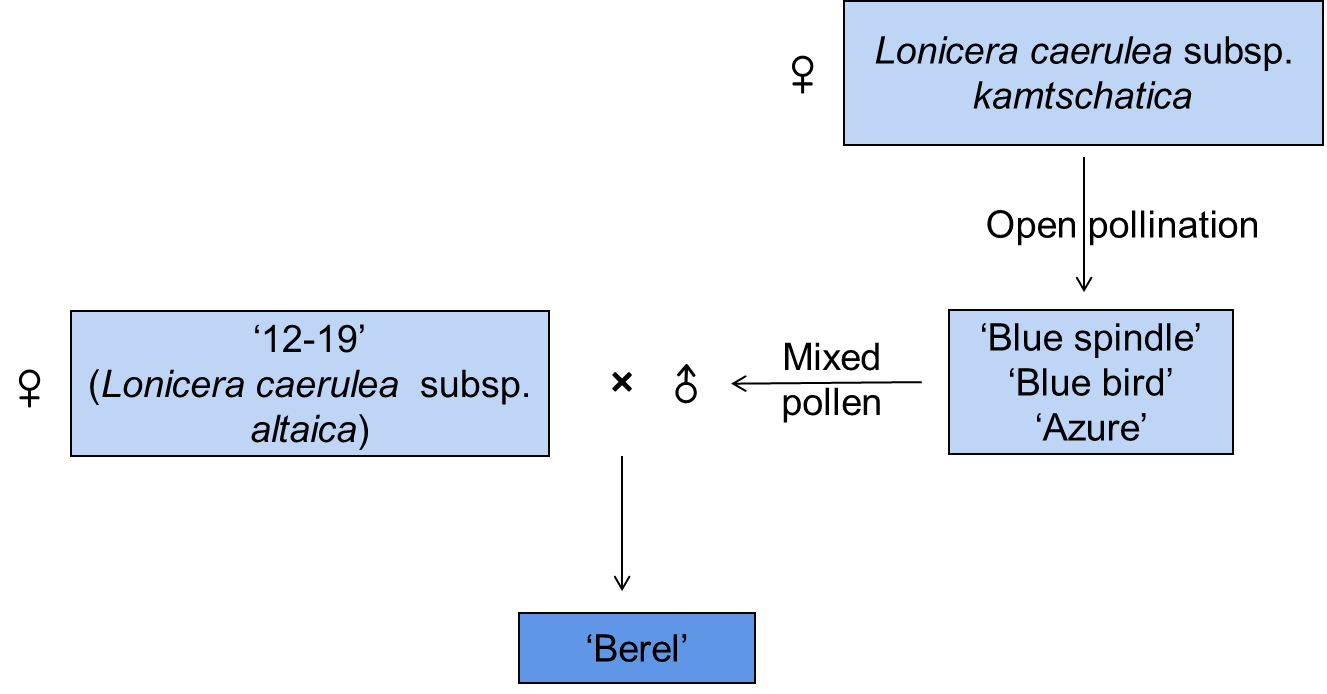
**

**Fig. S1** Pedigree of blue honeysuckle ‘Berel’.

**Table S1** Quantitation of phenolic compounds and antioxidant capacity of blue honeysuckle seeds and seed oil.

|  | Seeds | Seed Oil |
| --- | --- | --- |
| Content | | |
| TPC (mg GAE/g DW) | 37.40 ± 0.52^a^ | 3.16 ± 0.11^b^ |
| TFC (mg CE/g DW) | 8.52 ± 0.24^a^ | 0.30 ± 0.06^a^ |
| TAC (mg C3GE/g DW) | 27.29 ± 0.36 | - |
| TPAC (mg C3GE/g DW) | 0.073 ± 0.02 | - |
| Antioxidant capacity | | |
| DPPH (μmol TE/g DW) | 220.44 ± 12.14^a^ | 19.67 ± 0.32^b^ |
| FRAP (μmol Fe^2+^E/g DW) | 308.96 ± 10.63^a^ | 39.75 ± 3.80^b^ |
| ABTS (μmol TE/g DW) | 4.75 ± 0.20^a^ | 0.84 ± 0.03^a^ |

**Table S2** Proximate analysis of blue honeysuckle seeds

| Constituents | Value |
| --- | --- |
| Moisture (%) | 6.38 ± 0.11 |
| Crude protein (%) | 12.87 ± 0.79 |
| Crude fat (%) | 13.03 ± 1.03 |
| Ash (%) | 3.14 ± 0.01 |
| Dietary fiber (%) | 43.78 ± 3.11 |
| Carbohydrate (%) | 20.79 ± 1.59 |
| Shell to kernel ratio | 53:47 |
